# Increased Risk of Pre-eclampsia After Frozen-Thawed Embryo Transfer in Programming Cycles

**DOI:** 10.3389/fmed.2020.00104

**Published:** 2020-04-08

**Authors:** Ze Wang, Hong Liu, Haixia Song, Xiufang Li, Jingjing Jiang, Yan Sheng, Yuhua Shi

**Affiliations:** ^1^Center for Reproductive Medicine, Shandong University, Jinan, China; ^2^Shandong Provincial Clinical Medicine Research Center for Reproductive Health, Jinan, China; ^3^Department of Reproductive Medicine, Shanxi Provincial People's Hospital, Taiyuan, China

**Keywords:** hypertensive disorders in pregnancy, pre-eclampsia, corpus luteum, programming cycles, frozen-thawed embryo transfer

## Abstract

**Objective:** This study aims to investigate whether obstetric complications and perinatal outcomes after frozen embryo transfer (FET) in the programming cycles differ from that in the natural cycles.

**Methods:** We conducted a retrospective cohort study collecting a total of 14,373 singletons born after frozen embryo transfer at the Center for Reproductive Medicine Affiliated to Shandong University from September 2013 to September 2018. The women were divided into two groups according to the regimens for endometrium preparation: either natural cycles (*n* = 10,211) or programming cycles (*n* = 4,162). The primary outcomes were the incidence of obstetric complications consisting of pre-eclampsia, gestational diabetes mellitus, placenta previa, placental abruption, and postpartum hemorrhage. The perinatal outcomes included average birthweight, low birthweight (LBW), very LBW, macrosomia, large for gestational age, and small for gestational age. Multivariable logistic regression analysis was performed to adjust for potential confounders.

**Results:** The incidences of pre-eclampsia (8.6 vs. 3.8%) and postpartum hemorrhage (0.7 vs. 0.2%) in the programming FET cycles were significantly higher than those in the natural FET cycles. The logistic regression analysis showed that, compared to the natural FET cycles, the programming FET cycles were associated with an elevated risk of pre-eclampsia (aOR, 2.55; 95% CI, 2.06–3.16) and postpartum hemorrhage (aOR, 2.94; 95% CI, 1.44–5.99).

**Conclusion:** The women with singleton delivery after frozen-thawed embryo transfer in the programming cycles had an elevated risk of pre-eclampsia and postpartum hemorrhage, which was speculated to be associated with the absence of the corpus luteum.

## Introduction

In recent years, the freeze-all strategy of all available embryos has become a trend owing to its promising efficacy of maintained or even improved live birth rate, along with a decreased risk of developing ovarian hyperstimulation syndrome (OHSS) (1–3). Nevertheless, the mechanism underlying the finding of increased incidences of hypertensive disorders of pregnancy (HDP) and birth of larger for gestation age following frozen embryo transfer (FET), as reported by previous studies (3–6), remains unclear.

The widespread adoption of the freeze-all strategy into clinical practice enables the optimal cycle regimen of endometrial preparation for transfer of frozen-thawed embryos to be rethought (7), which reached no consensus so far. Basically, there are three options for the preparation of endometrium to reach an acceptable state for embryo implantation: natural ovulatory cycle, stimulated cycle, and programming cycle with hormonal replacement, for which the choice mainly depends on the physicians' discretion and the patients' preference. Detection of ovulation is the indicator for the timing of embryo thawed and transferred in the natural cycle or stimulated cycle, while the programming cycle is prepared by hormonal replacement to create a physiologic uterine receptivity for embryo implantation and thus being flexible to schedule the timing of embryo thawed and transferred. Scheduling is of convenience for patients to reduce the frequency of monitoring, as well as for clinicians and laboratory staff on weekdays.

The existing evidence comparing different regimens for endometrial preparation showed no significant difference in the rate of implantation, pregnancy, and live birth after FET (8), while only a few studies directly involved the obstetric and the neonatal outcomes. Notably, recent observational studies (9, 10) reported a higher risk of HDP among women who received FET prepared by the programming regimen as compared with that in FET cycles using the natural or stimulated regimens as well as fresh cycles or spontaneous conception. A possible mechanism might be explained by the absence of corpus luteum in the programming cycle, due to the suppression of ovulation. It was supposed that despite the replacement of luteal support by exogenous estrogen and progesterone in the programming cycles, some vasoactive substances produced by the corpus luteum after natural ovulation might play a key role in the maternal vascular health and subsequent placental development, suggesting that the absence of the corpus luteum or a less “physiological” status in the programming cycles may be a factor predisposing pregnant women to adverse obstetrical outcomes, such as pre-eclampsia (11–13).

The aim of this study was to investigate the effect of different regimens for endometrium preparation in FET on obstetric complications and perinatal outcomes, by the comparison of programmed cycles versus natural cycles.

## Materials and Methods

This study retrospectively collected singleton deliveries after frozen blastocyst transfer in either natural or programming cycles at the Center for Reproductive Medicine Affiliated to Shandong University, from September 2013 to September 2018. Women were excluded from this study if they (i) were more than 40 years old in age, (ii) had a body mass index (BMI) of 35 kg/m^2^ or greater, (iii) were diagnosed with polycystic ovary syndrome (PCOS), (iv) had a self-history of pre-eclampsia or a family history of pre-eclampsia, (v) were diagnosed with hypertension, diabetes, renal disease, or abnormal renal function, (vi) had a history of failure to obtain clinical pregnancy after an effort for more than three times of embryo transfer, and (vii) underwent frozen-thawed oocyte cycles. This study was approved by the independent ethics committee of the Center for Reproductive Medicine Affiliated to Shandong University.

For the natural cycle regimen in routine practice, the dominant follicle was monitored by transvaginal ultrasound until ovulation was achieved with or without the trigger of human chorionic gonadotrophin. Progesterone, at a dose of 20–30 mg daily, was administered for luteal phase support after ovulation, followed by the embryos being thawed and transferred on the fifth day after ovulation. The luteal support continued up to 10 weeks of gestation if pregnancy has been achieved. For the programming cycle regimen, estrogen, at a dose of 4–6 mg daily, was initiated on the second or third day of the menstrual cycle and lasted for 10–14 days commonly, with the purpose of promoting endometrial proliferation and inhibiting follicular growth. The dosage and duration of estrogen were raised until the endometrial thickness reached a proper state for embryo transfer (commonly at least 8 mm), at which time luteum support was added. Frozen blastocysts were thawed and transferred on the fifth day after progesterone initiation. In addition, serum luteinizing hormone (LH) and progesterone levels were measured to confirm whether luteinization existed. If conception was confirmed, estrogen was continued until 7 weeks of gestation, and progesterone was continued until 10 weeks of gestation. Follow-up for pregnant women was scheduled by telephone interview and information on obstetric and neonatal complications was obtained from medical records.

The primary outcomes were the incidences of obstetric complications consisting of pre-eclampsia, gestational diabetes mellitus (GDM), placenta previa, placental abruption and postpartum hemorrhage. The diagnosis of pre-eclampsia and GDM was based on correspondingly recommended guidelines. The mode for delivery, gestational age at delivery, preterm labor, and post-term birth were also compared between the two groups. Perinatal outcomes included average birthweight, low birthweight (LBW, <2,500 g), very LBW (<1,500 g), macrosomia (>4,000 g), large for gestational age (LGA), and small for gestational age (SGA). LGA and SGA were defined as birthweight higher than the 90th percentile and lower than the 10th percentile of referential birthweight for Chinese newborns, respectively, adjusted for gestational age and sex. Baseline characteristics included age, blood pressure, BMI, proportion of primary infertility, infertility diagnosis, endocrine measurements, and pregestational glucose screening results. Embryo transfer data included type of fertilization, number of embryos transferred, and endometrial thickness in FET.

### Statistical Analysis

Continuous variables were presented as mean and standard deviation with Student's *t*-test for the between-group differences. Categorical variables were expressed as frequencies and percentages, and the between-group differences were analyzed by chi-square test or Fisher's exact test when the expected frequencies were less than five. The crude and adjusted odds ratios with 95% confidence interval (CI) comparing the programming cycle versus the natural cycle for outcomes were performed by multivariable logistic regression, adjusted by maternal age, blood pressure, BMI, proportion of primary infertility, infertility diagnosis, number of antral follicle count (AFC), endocrine measurements and pregestational glucose level, type of fertilization, number of embryos transferred, and endometrial thickness in FET. All analyses were performed using SPSS software (version 22.0), and a two-sided *P*-value < 0.05 was considered as statistically significant.

## Results

A total of 14,373 singletons born following FET were collected in this study, categorized into 10,211 natural cycles and 4,162 programming cycles. Table 1 shows the clinical characteristics of the study population. The baseline BMI, blood pressure, blood fasting glucose, serum LH and testosterone, and the number of AFC and embryos transferred were higher the programming cycle than that in the natural cycle, while the maternal age, serum follicle-stimulating hormone, and endometrial thickness in FET were lower. There was no between-group difference regarding the proportion of primary infertility, infertility diagnosis and type of fertilization.

**Table 1:** Baseline demographic and clinical characteristics of natural vs. programming cycle.

	**Natural cycle (*n* = 10, 211)**	**Programming cycle (*n* = 4,162)**	***P***
Maternal age (years)	30.93 ± 4.16	30.63 ± 4.14	<0.001
Paternal age (years)	31.46 ± 4.78	31.25 ± 4.81	0.020
Baseline systolic pressure (mmHg)	119.36 ± 11.40	120.40 ± 10.98	<0.001
Baseline diastolic pressure (mmHg)	77.45 ± 8.65	78.20 ± 8.02	<0.001
Baseline BMI (kg/m^2^)	22.68 ± 3.20	23.30 ± 3.41	<0.001
**Baseline BMI (%)**			
<18.5	700 (6.9)	231 (5.6)	<0.001
18.5–23.9	6,405 (62.7)	2,325 (55.9)	
24.0–27.9	2,382 (23.3)	1,190 (28.6)	
≥28	724 (7.1)	416 (10.0)	
Nulliparity (%)	5,757 (56.4)	2,312 (55.6)	0.363
**Cause of infertility (%)**			
Pelvic factor	7,043 (69.0)	2,872 (69.0)	0.102
Male factor	2,785 (27.3)	1,103 (26.5)	
Unexplained infertility	167 (1.6)	72 (1.7)	
Others	216 (2.1)	115 (2.8)	
Proportion of IVF (%)	7,196 (70.5)	2,967 (71.3)	0.330
Baseline FSH (IU/L)	6.58 ± 1.65	6.37 ± 1.63	<0.001
Baseline LH(IU/L)	4.96 ± 2.06	5.33 ± 2.67	<0.001
Baseline T (n/dL)	24.71 ± 11.54	27.03 ± 12.45	<0.001
AFC	13.85 ± 5.37	14.70 ± 6.06	<0.001
Baseline fasting glucose (mmol/L)	5.22 ± 0.50	5.27 ± 0.50	<0.001
Number of embryos transferred	1.16 ± 0.37	1.18 ± 0.39	0.005
Endometrial thickness in FET (cm)	1.02 ± 0.16	0.94 ± 0.14	<0.001

*BMI, body mass index; IVF, in vitro fertilization; FSH, follicle-stimulating hormone; LH, luteinizing hormone; T, total testosterone; AFC, number of antral follicle count; FET, frozen embryo transfer*.

The obstetric and perinatal outcomes are shown in Table 2. Women with programming cycles had an increased incidence of pre-eclampsia, postpartum hemorrhage, preterm labor and cesarean section as compared to those with natural cycles, while the incidences of GDM, placenta previa, placental abruption, macrosomia, LBW, LGA, and SGA were comparable between the two groups. When adjusting for clinical characteristics in the multivariable logistic regression model, the programming cycles were associated with an elevated risk of pre-eclampsia (aOR, 2.55; 95% CI, 2.06–3.16) and postpartum hemorrhage (aOR, 2.94; 95% CI, 1.44–5.99) (Table 3).

**Table 2:** Obstetric and perinatal outcomes of natural vs. programming cycle.

	**Natural cycle (*n* = 10,211)**	**Programming cycle (*n* = 4,162)**	***P***
Pre-eclampsia (%)	393 (3.8)	356 (8.6)	<0.001
GDM (%)	575 (5.6)	259 (6.2)	0.169
Placenta previa (%)	77 (0.8)	39 (0.9)	0.266
Placental abruption (%)	10 (0.1)	9 (0.2)	0.077
Postpartum hemorrhage (%)	17 (0.2)	29 (0.7)	<0.001
Cesarean section (%)	7,091 (69.4)	3,360 (80.7)	<0.001
Gestational age at delivery (weeks)	39.15 ± 1.55	39.10 ± 1.74	0.099
Preterm labor (%)	581 (5.7)	315 (7.6)	<0.001
Post-term birth (%)	26 (0.3)	14 (0.3)	0.399
Average birthweight (g)	3455.6 ± 500.6	3459.7 ± 537.5	0.673
Male sex of neonates (%)	5,440 (53.3)	2,227 (53.5)	0.800
Macrosomia (%)	1,032 (10.1)	433 (10.4)	0.594
LBW (%)	296 (2.9)	141 (3.4)	0.121
VLBW (%)	39 (0.4)	26 (0.6)	0.049
LGA (%)	1,987 (19.5)	855 (20.5)	0.139
SGA (%)	516 (5.1)	226 (5.4)	0.355

*GDM, gestational diabetes mellitus; LBW, low birthweight; VLBW, very low birthweight; LGA, large for gestational age; SGA, small for gestational age*.

**Table 3:** Crude and adjusted ORs of programming cycle against natural cycle for pre-eclampsia and postpartum hemorrhage.

**Outcomes**	**Crude OR (95% CI)**	***P***	**Adjusted OR (95% CI)**	***P***
Pre-eclampsia (%)	2.34 (2.02–2.71)	<0.001	2.55 (2.06–3.16)	<0.001
Postpartum hemorrhage (%)	4.21 (2.31–7.66)	<0.001	2.94 (1.44–5.99)	0.003

*OR, odds ratio; CI, confidence interval*.

*Adjusted by maternal age, blood pressure, BMI, proportion of primary infertility, infertility diagnosis, number of antral follicle count, baseline FSH, LH, T, and fasting glucose level, type of fertilization, number of embryos transferred, and endometrial thickness in FET*.

## Discussion

This study confirmed that the programming cycle regimen for FET was an independent risk factor of pre-eclampsia and postpartum hemorrhage, whereas the incidences of GDM, placenta previa, placental abruption, macrosomia, LGA, and SGA were similar between the programming cycle and the natural cycle.

Our results were consistent with recent studies (9, 10) with limited sample size, suggesting an association between increased risk of pre-eclampsia and programming FET cycles. The possible mechanism was focused on the absence of the corpus luteum (12) in the programming cycles with suppression of ovulation; as evidenced by that, the lack of circulating vasoactive substances produced solely from the corpus luteum, such as relaxin (11, 13), may contribute to the impaired arterial compliance during early gestation and consequently an increased risk of HDP. Subsequently, in a large population-based registry study in Sweden (14), an elevated risk of HDP, postpartum hemorrhage, post-term birth, and macrosomia was detected in the programming FET cycles, while the stimulated cycles had the same outcomes as the natural cycles. In parallel, another large retrospective study of over 100,000 FET cycles in Japan (15) showed that, in addition to HDP, the risk of placenta accreta was higher while the risk of GDM was lower in the programming cycles as compared with that in the natural cycles in FET.

As a common condition complicating pregnancy, gestational hypertension and its progressing stage, pre-eclampsia, can lead to significant maternal and fetal morbidity. Despite unclear pathogenesis, several clinical risk factors have been reported to be strongly associated with the incidence of pre-eclampsia (16, 17), such as advanced maternal age, obesity, current condition of chronic hypertension, diabetes and renal disease, nulliparity, multiple gestation, prior pre-eclampsia, antiphospholipid antibody syndrome and assisted reproductive techniques. As reported by prior studies, our study supported the finding of increased risk of pre-eclampsia and postpartum hemorrhage, whereas the finding of decreased risk of GDM as well as elevated risk of post-term birth and macrosomia did not occur after excluding the presence of PCOS and controlling for clinical risk factors. We noticed that some clinical characteristics, such as BMI and the presence of PCOS, were unavailable in the study of Japan (15). Notably, PCOS is considered as a common reason driving the choice of programming cycles owing to internal ovulation dysfunction. It was unknown whether the finding of decreased risk of GDM in the programming cycle might be explained by a greater proportion of women with obesity and PCOS, since both of them were closely related with an increased risk of obstetric and neonatal complications. The birth of macrosomia is relevant to the incidence of post-term birth. We found that the incidence of post-term birth in the study in Sweden was 8.9% in the programming cycle compared with 5.8% in the natural cycle, whereas the proportion of post-term birth in our result was only merely 0.3% for both groups and, correspondingly, the proportion of cesarean section was 69.4% in the natural cycle and 80.7% in the programming cycle, which was 2-fold higher than that in the study in Sweden.

The transfer of frozen-thawed embryos has become the main modality of *in vitro* fertilization (IVF) cycles, primarily owing to the rapid development of laboratory techniques and favorable prognosis for freeze-all strategy (1). With single blastocyst transfer being popular and routinely applied in clinical practice, powerful evidence has shown that a dramatically increased live birth rate could be achieved by elective frozen blastocyst transfer (3). Nevertheless, an increased risk of maternal HDP and neonatal birth of LGA following FET warrants attention. It is plausible that this increase may be, to some extent, attributable to the absence of the corpus luteum and correspondingly attenuated maternal circulatory adaptation for pregnancy in the programming FET cycles. Our finding, in line with prior findings, timely calls for the rethought of optimal regimen for endometrial preparation given the increasing utilization of FET in IVF cycles, of which natural cycles ought to be encouraged for ovulatory women. However, it could be unadvisable to deny programming cycles, especially for women with polycystic ovary syndrome, considering a high risk of OHSS and multiple dominant follicle accompanied with stimulated cycles. A larger cohort or randomized controlled trial is required to confirm the association between adverse obstetric outcomes and the programming cycles without a corpus luteum. Whether there are other potential factors responsible for the finding of increased risk of HDP in FET or whether a substitution for the deficiency of corpus luteum products in the programming cycles might improve the maternal and neonatal health warrants further research.

Data on gestational hypertension are not available, which is a limitation of our study. Besides, women with fresh cycles or stimulated FET cycles were not collected into this study, where one or more corpus luteum exist. This information may provide a supplementary support for the hypothesis of a link between adverse maternal outcomes and absence of corpus luteum in the programming FET cycles. Additionally, data on obstetric complications were incomplete because women with pregnancy loss in the second or the third trimester were not included in this study.

In conclusion, women with singleton delivery after frozen-thawed embryo transfer in the programming cycles had an elevated risk of pre-eclampsia and postpartum hemorrhage, which was speculated to be associated with the absence of the corpus luteum. For ovulatory women, it is suggested that the natural cycles ought to be recommended as priority for the endometrial preparation of FET. Future prospective studies should be conducted.

## Data Availability Statement

All datasets generated for this study are included in the article/supplementary material.

## Author Contributions

YShe and YShi contributed to the study concept and design of this study. ZW, HL, and HS contributed to the acquisition, analysis, interpretation of data, and the drafting of the paper. XL and JJ contributed to the review and the revision of the manuscript. All authors give final approval to this manuscript for publication.

### Conflict of Interest

The authors declare that the research was conducted in the absence of any commercial or financial relationships that could be construed as a potential conflict of interest.
